# Effect of Osteoprotegerin and Dickkopf-Related Protein 1 on Radiological Progression in Tightly Controlled Rheumatoid Arthritis

**DOI:** 10.1371/journal.pone.0166691

**Published:** 2016-12-02

**Authors:** Carmen Gómez-Vaquero, Irene Martín, Estibaliz Loza, Loreto Carmona, José Ivorra, José Antonio Narváez, Javier Hernández-Gañán, Pedro Alía, Javier Narváez

**Affiliations:** 1 Department of Rheumatology. Hospital Universitari de Bellvitge. Barcelona, Spain; 2 Instituto de Salud Músculoesquelética. Madrid, Spain; 3 Department of Rheumatology. Hospital Universitario La Fe. Valencia. Spain; 4 Department of Radiology. Hospital Universitari de Bellvitge. Barcelona, Spain; 5 Department of Clinical Laboratory. Hospital Universitari de Bellvitge. Barcelona, Spain; Nippon Medical School Hospital, JAPAN

## Abstract

**Objective:**

To analyze the association between circulating osteoprotegerin (OPG) and Dickkopf-related protein 1 (DKK-1) and radiological progression in patients with tightly controlled rheumatoid arthritis (RA).

**Methods:**

Serum levels of OPG and DKK-1 were measured in 97 RA patients who were treated according to a treat-to-target strategy (T2T) aimed at remission (DAS28<2.6). Radiologic joint damage progression was assessed by changes in the total Sharp-van der Heijde score (SHS) on serial radiographs of the hands and feet. The independent association between these biomarker levels and the structural damage endpoint was examined using regression analysis

**Results:**

The mean age of the 97 RA patients (68 women) at the time of the study was 54 ± 14 years, and the median disease duration was 1.6 ± 1.5 years. Most patients were seropositive for either RF or ACPA, and the large majority (76%) were in remission or had low disease activity.

After a median follow-up time of 3.3 ± 1.5 years (range, 1–7.5 yrs.), the mean total SHS annual progression was 0.88 ± 2.20 units. Fifty-two percent of the patients had no progression (defined as a total SHS of zero). The mean serum OPG level did not change significantly over the study period (from 3.9 ± 1.8 to 4.07 ± 2.23 pmol/L), whereas the mean serum DKK-1 level decreased, although not significantly (from 29.9 ± 10.9 to 23.6 ± 18.8 pmol/L).

In the multivariate analysis, the predictive factors increasing the likelihood of total SHS progression were age (OR per year = 1.10; *p* = 0.003) and a high mean C-reactive protein level over the study period (OR = 1.29; *p* = 0.005). Circulating OPG showed a protective effect reducing the likelihood of joint space narrowing by 60% (95% CI: 0.38–0.94) and the total SHS progression by 48% (95% CI: 0.28–0.83). The DKK-1 levels were not associated with radiological progression.

**Conclusion:**

In patients with tightly controlled RA, serum OPG was inversely associated with progression of joint destruction. This biomarker may be useful in combination with other risk factors to improve prediction in patients in clinical remission or low disease activity state.

## Introduction

In rheumatoid arthritis (RA), remission or low disease activity can be achieved with tight control of inflammation and early use of disease-modifying antirrheumatic agents (DMARD). The importance of the treat-to-target strategy (T2T) has recently been highlighted by EULAR recommendations [1,2]. However, the definitions of remission according to clinical criteria, including disease activity score (DAS), simplified disease activity index (SDAI), and ACR/EULAR Boolean criteria do not always correspond with the complete absence of inflammation as measured by sensitive imaging techniques, such as magnetic resonance imaging (MRI) or ultrasonography (US) [3–6]. Several studies have demonstrated the presence of subclinical inflammation in a significant number of patients who were considered to be in clinical remission or at a low state of disease activity [3,6–8]. This persistent subclinical joint activity ultimately lead to radiographic joint damage progression [3,6–8].

Several predictors of clinical outcome and radiographic progression have been proposed in RA, including traditional inflammatory markers (ESR and C-reactive protein), patient´s characteristics, and genetic, serologic and imaging biomarkers [9–12]. Among serological biomarkers, recent works have suggested that some bone remodeling markers may be independent predictors of joint damage in RA [9,13–15]. If the level of a bone remodeling biomarker or, particularly the short-term change in the level, may predict radiographic progression, these markers may constitute disease activity indicators and may also be useful for clinicial managing of individual patients.

The characteristic trait of RA is a persistent inflammation of the synovial membrane and the formation of an invasive synovial tissue, called the pannus, that invades and destroys the adjacent cartilage and subchondral bone. The Receptor Activator of Nuclear Factor Kappa B Ligand (RANKL), osteoprotegerin (OPG) and Dickkopf-1 (DKK-1) have been demonstrated to be key molecules involved in bone erosion and bone remodeling [16,17]. The aim of the present study was to test whether these three bone remodeling biomarkers may serve as predictors of radiographic progression in patients with tightly controlled RA.

## Methods

### Study population

An observational longitudinal prospective study was carried out. A total of 97 patients with RA meeting the 2010 classification criteria for RA [18] were included. All patients were treated in the Early Arthritis Clinic of Bellvitge Hospital by the same rheumatologist (JN). They were treated according to a treat-to-target strategy (T2T) aimed at remission (DAS28 < 2.6). Patients were initially managed with a single synthetic DMARD, mainly methotrexate (MTX) or leflunomide (LEF), followed by a synthetic DMARD combination (usually MTX and LEF), and an exchange of LEF with biologic agents in case of failure.

The study was approved by the Clinical Research Ethics Committee of Bellvitge University Hospital-IDIBELL; Ref:PR/16511). All patients provided a written informed consent before participating in the study. The patient´s clinical records and information were anonymized and de-identified prior to analysis. This study was conducted in accordance with the principles of the Declaration of Helsinki and the International Conference for Harmonization.

### Clinical and laboratory profiles

Radiographs of hands, wrists, and feet were obtained at inclusion of the study and after a minimum follow-up of 1 year (median ± standard deviation: 3.3 ± 1.5 years; range, 1–7.5 yrs). In this study, time zero (T0) refers to the baseline blood and radiologic examinations, and time one (T1) to the date of the second radiograph and blood sample. Radiographs were digitalized and scored for erosions (ERO) and joint space narrowing (JSN) using the Sharp-van der Heijde score (SHS). The images were independently reviewed by three of the authors (JI, JAN and JHG) who were blinded to the clinical information and the date of Rx acquisition. Discrepancies in findings were resolved in a subsequent joint review session and consensus was reached.

The serum OPG, DKK-1 and soluble RANKL levels were measured by commercial ELISA kits (Biomedica® Immunoassays, Vienna, Austria) at T0 and T1. Consistent with the kit manufacturer’s instructions [19], samples were immediately centrifuged and stored at -20°C until they were assayed. Our initial intention was to evaluate the ratio RANKL / OPG, but the RANKL values were below the detection limit in 82 (85%) patients. Therefore, in our study, we ultimately only tested OPG and DKK-1. According to the manufacturers, normal values are below 2.7 pmol/l for OPG and 34 pmol/l for DKK-1.

The primary outcome measure of the study was the presence of radiographic progression, which was defined as any increase in the total SHS between baseline (T0) and the second radiograph (T1). No progression was defined as a score of zero. The minimal clinically important difference was defined as an increase of at least 5 points in total SHS.Outpatient charts were comprehensively reviewed following a specifically designed protocol. Baseline data collected at T0 included the following: age, gender, body mass index (BMI), disease duration (calculated from data of the first symptom), presence of extra-articular manifestations, details of past and present anti-rheumatic therapies (NSAID, steroids, DMARD and the number of biological agents previously used) and osteoporosis treatments, erythrocyte sedimentation rate (ESR), C-reactive protein (CRP), and baseline serological status for rheumatoid factor (RF) and anti-citrullinated peptide antibodies (ACPA). ACPA antibodies were measured using a commercially available second-generation ELISA kit (EIA^TM^ ACPA Assay on the ImmunoCAP250 instrument, Phadia, Germany).

In addition, there was an assessment of disease activity at T0 that included the patient´s visual analog scale for pain, the swollen and tender joint count in 28 joints, the DAS28-ESR score, and the health assessment questionnaire (HAQ). As a reflection of articular disease activity between T0 and T1, we also calculated the mean DAS28-ESR and the mean CRP of all of the values recorded in the monitoring visits conducted during this period of time.

### Statistical analysis

The sample was described by using summary statistics, i.e., the mean, median, standard deviation, and interquartile range for quantitative variables and distribution percentages for qualitative variables.

Crude associations between the presence of radiographic progression expressed in a dichotomous variable (progression / no progression) and clinical and laboratory variables were investigated by bivariate logistic regression models, expressing the results as an odds ratios (OR) and significance value.

A multivariate logistic regression analysis was performed to assess the predictive role of the OPG and DKK-1 levels on radiological progression, controlling for potential confounders such as age, sex, disease activity, mean corticosteroid dose, and DMARD treatment duration. The construction of the regression models was perfomed by backward stepwise using both statistical and clinical judgment. The interaction effect of gender was tested by stratified analysis.

## Results

The main demographic and clinical characteristics of the RA study cohort are summarized in Table 1.

**Table 1:** Main demographic and clinical characteristics of the RA study cohort.

**Number of patients**	97
**Women/men**	68 (70%) / 29 (30%)
**Age, years**	53 ±14
**BMI, kg/m**^**2**^	25.9 ± 4.9
**Disease duration (median), years**	1.6 ± 1.5
**Positive RF**	59 (61%)
**Positive ACPA**	60 (62%)
Systemic extraarticular manifestations^*^	16 (16%)
**DAS28-ESR at T0*Baseline disease activity at T0***	3.4 ± 1.8
* • Remission or low activity disease*	71 (76%)
* • Moderate*	21 (23%)
* • High*	1 (1%)
**HAQ (0–3)**	1.38 ± 0.88
**ESR (mm/h) at T0**	16.7 ± 13.4
**CRP (mg/L) at T0**	9.6 ± 16.2
**OPG (pmol/L) at T0**	3.9 ± 1.8
**DKK-1 (pmol/L) at T0**	29.9 ± 10.9
**Follow-up time between T0 and T1, (median) years**	3.3 ± 1.5 (range: 1–7.5)
**Treatment during follow-up period*** • Synthetic DMARD monotherapy*	62 (64%)
* • Synthetic DMARD combinations*	15 (15%)
* • Biological therapy + synthetic DMARD*	20 (21%)
• Concomitant therapy ○ ***Low-dose oral glucocorticoid treatment***	69 (72%)
○ *Accumulated dose of prednisone (g)*	4.28 ± 2.86
**Osteoporosis treatment**	
* • None*	26 (28%)
* • Calcium and Vitamin D*	70 (72%)
* • Antiresorptive or bone forming therapy*	36 (37%)
**Mean DAS28-ESR between T0 and T1**	2.6 ± 0.95
**Mean CRP between T0 and T1**	2.48 ± 0.87
**Radiological progression (annual difference)*** • Erosions*	0.19 ± 0.62
* • Joint space narrowing*	0.68 ± 1.70
* • Total Sharp–van der Heijde score*	0.88 ± 2.20

Results are presented as mean ± standard deviation (median for disease duration and follow-up time) or number of cases with percentages.

*Including rheumatoid nodules, pulmonary manifestations, vasculitis, ocular manifestations, and secondary Sjögren’s syndrome.

**Abbreviations:** ACPA = anti-citrullinated peptide antibodies; BMI = body mass index; CRP = C-reactive protein; DAS = disease activity score; DKK-1 = Dickkopf-related protein 1; DMARD = disease modifying anti-rheumatic drug; ESR = erythrocyte sedimentation rate; HAQ = Health Assessment Questionnaire; OPG = osteoprotegerin.

The mean age of the 97 RA patients (68 women) at the time of the study was 54 ± 14 years, and the median disease duration was 1.6 ± 1.5 years. Most patients were seropositive for either RF or ACPA, and the large majority (76%) were in remission or had low disease activity.

The median follow-up time between T0 and T1 was 3.3 ± 1.5 years (range, 1–7.5 yrs.). Of the 97 patients, 62 (64%) received synthetic DMARD monotherapy during the follow-up period, 15 (15%) received synthetic DMARD combinations, and 20 (21%) ultimately required biological therapy (12% were taking a TNF inhibitor, 5% rituximab and 4% tocilizumab). Additionally, 69 (72%) patients also received a concomitant low dose oral glucocorticoid treatment, and 36 (37%) were received antiresorptive or bone-forming therapy (34 with bisphosphonates, 1 with denosumab and 1 with teriparatide).

The mean DAS28-ESR value during the follow-up period was 2.6 ± 0.9, and the mean CRP value was 2.48 ± 0.87 (reference value < 5 mg/L). The mean serum OPG level did not change significantly over the study period (from 3.9 ± 1.8 to 4.07 ± 2.23 pmol/L), whereas the mean serum DKK-1 level tended to decrease (from 29.9 ± 10.9 to 23.6 ± 18.8 pmol/L; *p* = NS. There were not differences in DKK1 serum levels between patients treated with TNF inhibitors and patients not treated with biological agents or treated with rituximab or tocilizumab.

The mean total SHS annual progression over the study period (see Table 1) was 0.88 ± 2.20 units (below the minimal clinically important difference). Fifty two percent (50) of the patients had no progression.

Table 2 shows the results of a bivariate analysis of factors associated with radiographic progression. The annual ERO score progression was associated with a longer follow-up time T0-T1, longer duration of synthetic DMARD therapy, and with greater changes in the mean DAS28-ESR and mean CRP between T0 and T1. The age, BMI, and high CRP levels over the study period were all associated with the increased probability of JSN progression (6%, 12% and 8%, respectively). Finally, age, BMI, and the higher values of the mean DAS28-ESR and mean CRP levels over the follow-up period were associated with total SHS progression. Of note, in this analysis, neither the OPG nor DKK-1 serum levels achieved statistical significance.

**Table 2:** Factors associated with radiographic progression by the Sharp-van der Heijde score (total and by subscales): results of the bivariate analysis.

Variable	Erosion OR (p-value)	Joint space narrowing OR (p-value)	Total score OR (p-value)
**Female sex**	0.60 (0.286)	0.73 (0.502)	0.77 (0.569)
**Age, per year**	1.02 (0.165)	1.06 (0.002)	1.05 (0.003)
**BMI, per unit**	1.01 (0.881)	1.12 (0.026)	1.11 (0.039)
**Disease duration, per year**	0.99 (0.960)	0.94 (0.656)	0.99 (0.926)
**Follow-up time T0-T1, per year**	1.61 (0.005)	0.97 (0.832)	1.05 (0.712)
**Positive RF**	1.29 (0.596)	1.63 (0.279)	1.53 (0.330)
**Positive ACPA**	1.00 (0.992)	0.53 (0.148)	0.63 (0.289)
**Mean DAS28-ESR between T0 and T1, per unit**	1.99 (0.008)	1.41 (0.124)	1.88 (0.009)
**Mean CRP between T0 and T1, per unit**	1.16 (0.001)	1.08 (0.024)	1.20 (0.002)
**OPG1, per pmol /L**	0.94 (0.660)	1.04 (0.768)	1.02 (0.866)
**DKK-1, per pmol /L**	1.02 (0.352)	1.00 (0.959)	1.01 (0.548)
**Accumulated glucocorticoid dose, per mg**	1.00 (0.180)	1.00 (0.442)	1.00 (0.165)
**Duration of synthetic DMARD therapy, per month**	1.04 (0.007)	1.00 (0.983)	1.00 (0.724)
**Anti-TNFα treatment**	0.60 (0.209)	0.88 (0.570)	0.84 (0.417)
**Antiresorptive or bone forming therapy**	0.65 (0.256)	0.96 (0.737)	0.94 (0.636)

**Abbreviations**: ACPA = anti-citrullinated peptide antibodies; BMI = body mass index; CRP = C-reactive protein; DAS = disease activity score; DKK-1 = Dickkopf-related protein 1; DMARD = disease modifying anti-rheumatic drug; ESR = erythrocyte sedimentation rate; OPG = osteoprotegerin; OR = odds ratio; RF = rheumatoid factor; T0 = baseline visit; T1 = date of the second radiograph.

On multivariate, no significant differences in the role of the different independent variables on the basis of sex were observed. In the multivariate analysis (Table 3), ERO score progression was associated with a longer follow up time T0-T1 and high mean CRP levels over the study period. JSN and total SHS progression increased with age and also with the higher values of the mean CRP between T0 and T1. Circulating OPG showed a protective effect reducing the likelihood of JSN by 60% (OR: 0.60, 95% CI: 0.38–0.94) and the total SHS progression by 48% (OR: 0.48, 95% CI: 0.28–0.83). The DKK-1 levels were apparently not associated with radiological progression.

**Table 3:** Factors associated with radiographic progression by the Sharp-van der Heijde score (total and by subscales): results of the multivariate regression models by backward stepwise selection.

	Erosion OR (p-value)	Joint space narrowing OR (p-value)	Total score OR (p-value)
**Female sex**	NS	NS	NS
**Age, per year**	NS	1.10 (0.004)	1.10 (0.003)
**Mean CRP between T0 and T1, per unit**	1.18 (0.001)	1.08 (0.047)	1.29 (0.005)
**Follow-up time T0-T1, per year**	1.61 (0.025)	NS	NS
**Mean DAS28-ESR between T0 and T1, per unit**	NS	NS	NS
**OPG1, per pmol /L**	NS	0.60 (0.026)	0.48 (0.008)
**DKK-1, per pmol /L**	NS	NS	NS
**Accumulated glucocorticoid dose, per mg**	NS	NS	NS
**Duration of synthetic DMARD therapy, per month**	NS	NS	NS
**Anti-TNFα treatment**	NS	NS	NS

**Abbreviations**: CRP = C-reactive protein; DAS = disease activity score; DKK-1 = Dickkopf-related protein 1; DMARD = disease modifying anti-rheumatic drug; ESR = erythrocyte sedimentation rate; OPG = osteoprotegerin; OR = odds ratio; T0 = baseline visit; T1 = date of the second radiograph.

Erosion: Pseudo R^2^ = 0.25; Space narrowing: Pseudo R^2^ = 0.17; Total score: Pseudo R^2^ = 0.33

Neither anti-TNFα therapy or the antiresorptive or bone-forming therapy influenced in the OPG/DKK1 levels nor in the radiographic progression.

## Discussion

One of the hallmarks of RA is progressive bone erosion. In this entity, erosion of periarticular cortical bone results from osteoclastic bone resorption at the site of synovitis, where RANKL expression is found [16]. RANKL is a membrane protein that is secreted by osteoblasts and osteocytes under the influence of proinflammatory cytokines, such as tumor necrosis factor-α (TNF-α), interleukin (IL)-1 and IL-6 [17]. The binding of RANKL to the RANK (Receptor Activator of Nuclear factor Kappa B) receptors on pre-osteoclasts and mature osteoclasts and stimulates their activation and differentiation, finally leading to enhanced bone resorption [13,16]. This bone resorption is inhibited by OPG, which prevents RANKL-RANK interactions [13,16]. Thus, the balance between RANKL and OPG regulates the degree of proliferation and activity of the osteoclasts, playing a pivotal role in bone erosion.

Crotti et al. showed that synovial RANKL expression is increased in RA patients with active disease compared with patients with quiescent disease [20]. The increased levels of RANKL in inflamed joints lead to a high RANKL/OPG ratio, reflecting bone destruction, which is predictive of increased radiological progression. In this sense, Van Tuyl et al. found that a high baseline RANKL/OPG ratio in patients with early, active untreated RA was a strong independent predictor of rapid and persistent damage progression over the 11-year follow-up in the COBRA study [21]. The results from the logistic regression analysis performed at 5 years of this study showed that a high RANKL level gave an OR of 4.4 (1.5–13.0) for progression and high OPG levels, an OR of 0.29 (0.10–0.85) [22]. These data are in agreement with our results, as we also found that serum OPG may have a protective effect on radiographic disease progression, reducing the likelihood of joint space narrowing by 60% and the total SHS progression by 48%. Previous studies have demonstrated that OPG is decreased in the synovium and serum of active RA patients [23]. By contrast, increased serum OPG was found after TNF-α inhibitor treatment in RA patients [24], in the same way that OPG expression is increased in the synovium of anti-TNF treated patients [25]. Furthermore, it was recently shown that genetic variants in OPG are associated with progression of joint destruction in RA [26].

In our study, our initial intention was to evaluate the RANKL / OPG ratio, but the RANKL values were below the detection limit in 85% of the patients. This may be explained by the fact that it was a cohort of RA patients treated according to treat-to-target strategy who mostly (76%) were in remission or had low disease activity at the time of the study. Several studies [27–29] have demonstrated that TNF-α inhibitors and some synthetic DMARDs (such as MTX and sulfasalazine) inhibit the expression of RANKL in RA synoviocytes while augmenting the secretion of OPG in synoviocyte supernatants, and they all inhibited osteoclast formation in vitro. In addition, we cannot forget that the accurate measurement of circulating RANKL is very difficult because of uncertain factors about which forms are the most biologically relevant and the limited sensitivity of available assays [9,13,14]. In this sense, Chan et al. showed significant (> 50%) alterations in serum concentration of RANKL after storage for 6 months at both -20°C and -70°C [30].

In addition to the OPG/RANK/RANKL pathway, several lines of evidence show that the Wnt signaling pathway also significantly participates in RA pathogenesis. DKK-1 is an endogenous, secreted inhibitory factor in canonical Wnt signaling by binding the Wnt coreceptor LRP5/6 [31]. DKK-1 is thought to be the main determinant of the uncoupling of bone remodelling in RA: bone resorption and bone formation are uncoupled in favor of resorption. In addition, studies have demonstrated that activation of the Wnt signaling pathway in mature osteoblasts upregulates OPG, which blocks RANKL-induced osteoclastogenesis and results in the inhibition of bone resorption [32,33]. When DKK-1 is increased by TNF-α, it exerts its negative regulation on the WnT pathway, decreasing the expression of OPG, enhancing the RANKL/RANK interaction, blocking osteoblast differentiation and inducing the expression of sclerostin, leading to the death of osteocytes [34]. DKK-1 also plays a key role in the promotion of synovial angiogenesis [35].

The serum levels of DKK-1 are significantly higher in patients with active RA than in healthy controls [15], and the change in the DDK-1 levels may serve as a biomarker of disease activity and radiographic progression [9,15,36]. Garnero et al. found that higher levels of DKK-1 are associated with an increased risk of articular erosions independent of age, baseline radiographic features, CRP or disease activity [36]. However, in our study, circulating DKK-1 was not an independent predictor of radiographic progression. This discrepancy can also be explained by the characteristics of our RA cohort, consisting of mostly patients with low disease activity or who were in remission. In this sense, it has been demonstrated that circulating DDK-1 decreases after treatment with TNF-α inhibitors or the IL-1 receptor antagonist anakinra [15,37], whereas the effect of synthetic DMARDs on DKK-1 production has not been specifically evaluated.

This study has strengths and limitations. The strengths are its prospective design, the analysis of the short-term change in the level of the biomarkers (not limited to baseline samples), and the fact that known confounders in the multivariate analyses are addressed. In addition, we evaluated for the first time the predictive value of bone remodeling biomarkers in patients with tightly controlled RA. The main limitations include the relatively small sample size, the variation in follow-up intervals for patients, and the evaluation of radiological progression performed under different therapeutics. Therefore, we cannot conclusively rule out the possibility of treatment confounders.

In conclusion, in patients with tightly controlled RA, serum OPG was inversely associated with the progression of joint destruction.

Contrary to our results and other previous reports [20–25], two recently published studies question this protective role. Audo et al [38] using data from the ESPOIR cohort showed that a high OPG/TRAIL (tumor necrosis factor-related apoptosis-inducing ligand) was associated with lack of disease remission in RA and with rapid progression of erosions. In the same line, Van Steenbergen et al [39] showed that low OPG levels are associated with achieving DAS remission as well as DMARD-free sustained remission in RA. These discrepancies may be related by the fact that beside its well-known role in bone metabolism, OPG has pro-inflammatory effects that likely act via the activation of the nuclear factor Kappa B pathway [40,41]. Another possible explanation for these divergences could be related by the differences between the populations studied (our study was performed in patients with tightly controlled RA).

In view of these contradictory results, further studies are required to clarify the relevance of OPG in disease/progression activity in RA, and to examine whether this biomarker may be useful in combination with other risk factors to improve predictions and guide treatment decisions.
